# Cytokine Levels Correlate with Immune Cell Infiltration after Anti-VEGF Therapy in Preclinical Mouse Models of Breast Cancer

**DOI:** 10.1371/journal.pone.0007669

**Published:** 2009-11-03

**Authors:** Christina L. Roland, Kristi D. Lynn, Jason E. Toombs, Sean P. Dineen, D. Gomika Udugamasooriya, Rolf A. Brekken

**Affiliations:** 1 Division of Surgical Oncology, Department of Surgery, Hamon Center for Therapeutic Oncology Research, University of Texas Southwestern Medical Center, Dallas, Texas, United States of America; 2 Advanced Imaging Research Center and Department of Biochemistry University of Texas Southwestern Medical Center, Dallas, Texas, United States of America; 3 Department of Pharmacology, University of Texas Southwestern Medical Center, Dallas, Texas, United States of America; Centre de Recherche Public de la Santé (CRP-Santé), Luxembourg

## Abstract

The effect of blocking VEGF activity in solid tumors extends beyond inhibition of angiogenesis. However, no studies have compared the effectiveness of mechanistically different anti-VEGF inhibitors with respect to changes in tumor growth and alterations in the tumor microenvironment. In this study we use three distinct breast cancer models, a MDA-MB-231 xenograft model, a 4T1 syngenic model, and a transgenic model using MMTV-PyMT mice, to explore the effects of various anti-VEGF therapies on tumor vasculature, immune cell infiltration, and cytokine levels. Tumor vasculature and immune cell infiltration were evaluated using immunohistochemistry. Cytokine levels were evaluated using ELISA and electrochemiluminescence. We found that blocking the activation of VEGF receptor resulted in changes in intra-tumoral cytokine levels, specifically IL-1β, IL-6 and CXCL1. Modulation of the level these cytokines is important for controlling immune cell infiltration and ultimately tumor growth. Furthermore, we demonstrate that selective inhibition of VEGF binding to VEGFR2 with r84 is more effective at controlling tumor growth and inhibiting the infiltration of suppressive immune cells (MDSC, Treg, macrophages) while increasing the mature dendritic cell fraction than other anti-VEGF strategies. In addition, we found that changes in serum IL-1β and IL-6 levels correlated with response to therapy, identifying two possible biomarkers for assessing the effectiveness of anti-VEGF therapy in breast cancer patients.

## Introduction

Virchow first identified a link between inflammation and cancer in the late 1800s [1]. Since that time, the concept that chronic inflammation in the tumor microenvironment contributes to tumor progression has been validated in many types of cancer [1], [2], [3]. However, the underlying mechanisms for this connection remain unclear. Solid tumor malignancies consist of a diverse population of cells, including tumor cells, fibroblasts, endothelial cells and immune cells [4], [5]. It is now clear that chronically activated immune cells can promote tumor growth and facilitate tumor survival. Macrophages are typically the main inflammatory component, but a variety of immune cells infiltrate tumors and can participate in tumor promotion [6]. In general, these cells confer a worse prognosis in many types of cancer, including breast cancer [7].

Vascular endothelial growth factor-A (VEGF) is a primary stimulant for tumor angiogenesis, making it a critical target for cancer therapy [8]. VEGF binds and activates VEGF receptor 1 (VEGFR1) and VEGFR2. Although the function of VEGFR2 in tumor angiogenesis has been characterized thoroughly, the function of VEGFR1 has not been well defined [9]. Clinically, elevated levels of VEGF correlate with increased lymph node metastases and a worse prognosis in breast cancer [10]. Bevacizumab (Avastin®, Genentech), a humanized monoclonal antibody that binds human VEGF and prevents VEGF from binding VEGFR1 and VEGFR2, is approved for the treatment of metastatic HER2/NEU-negative breast cancer [11]. The clinical success of bevacizumab has bolstered the development and testing of agents that directly target VEGF, selectively inhibit VEGFR1 or VEGFR2, or promiscuously block both VEGF receptors as well as other receptor tyrosine kinases [12], [13]. Previously, we have shown that selective inhibition of VEGF binding to VEGFR2 with a fully human monoclonal antibody (r84) is sufficient for effective control of tumor growth in a preclinical model of breast cancer [14]. However, few studies have compared directly the effectiveness of different anti-VEGF strategies in preclinical models.

The anti-tumor effect of angiogenesis inhibitors is due in part to reduction of VEGF-induced angiogenesis [15]. Immune cells also express receptors for VEGF; however, the effect of anti-VEGF therapy on the infiltration of immune cells into tumors has not been fully characterized. VEGF is a major chemoattractant for inflammatory cells, including macrophages, neutrophils, dendritic cells (DCs), myeloid-derived suppressor cells (MDSCs) and T-cells [16], [17], [18], [19], [20], [21]. In tumor xenograft models, anti-VEGF therapy leads to a reduction in macrophage infiltration [14], [16], [22], [23]. Recently, we found that selective inhibition of VEGF from binding VEGFR2 with r84 resulted in decreased in MDSC infiltration and increased neutrophil and mature dendritic cell infiltration in MDA-MB-231 human breast cancer xenografts [14]. Like macrophages, MDSCs (CD11b^+^Gr1^+^) are an important contributor to tumor progression whereby, these cells secrete immunosuppressive mediators and induce T-lymphocyte dysfunction [24], [25]. MDSCs express VEGFR1 and VEGFR2 [6] and studies in non-tumor bearing animals demonstrate that activation of VEGFR2 promotes MDSC infiltration into the spleen [17]. VEGF is also important for monocyte chemotaxis and is a key regulator of the differentiation and migration of dendritic cells (DCs) [17], [26]. In non-tumor bearing animals, VEGFR1 activation inhibits stem cell differentiation to the dendritic cell lineage whereas VEGFR2 activation decreases the number and function of mature dendritic cells in the spleen [17]. Unlike other myeloid cell types, increased tumor-infiltrating DCs is associated with improved prognosis and specifically, the number of CD83^+^ DCs has been shown to inversely correlate with lymph node metastasis and tissue expression of VEGF and TGF-β in human breast cancer specimens [27]. CD4^+^CD25^+^FoxP3^+^ regulatory T cells (Treg) contribute to maintenance of immunologic self-tolerance. However, the function of Treg as natural immune suppressors may also contribute to the immune imbalance found in cancers [28]. Clinically, blood from patients with breast or pancreatic cancer has an increased percentage of Treg compared to healthy individuals [29]. Treg secrete immunosuppressive cytokines such as TGF-β and IL-10, but little IFN-γ [29]. Though TGF-β can induce peripheral Treg, it is not required for the generation of a thymic-derived subset of these cells. Recently, IL-2, IFN-γ and TNF-α have been implicated in Treg generation [30], [31], [32]. However, the effect of anti-VEGF therapy on Treg infiltration is unknown.

In the present study, we use three distinct preclinical models of breast cancer to compare the effect of different anti-VEGF therapies on breast cancer growth, vascular parameters, immune cell infiltration and intra-tumoral cytokine levels. We found that inhibition of VEGF receptor activation resulted in changes in intra-tumoral levels of IL-1β and CXCL1 that correlate with changes in immune cell infiltration. Furthermore, serum levels of IL-1β and IL-6 correlate with tumor response to anti-VEGF therapy and may be predictive clinical markers.

## Results

### Comparison of Anti-VEGF Strategies on MDA-MB-231 Tumor Growth and Angiogenesis

**Figure 1:**
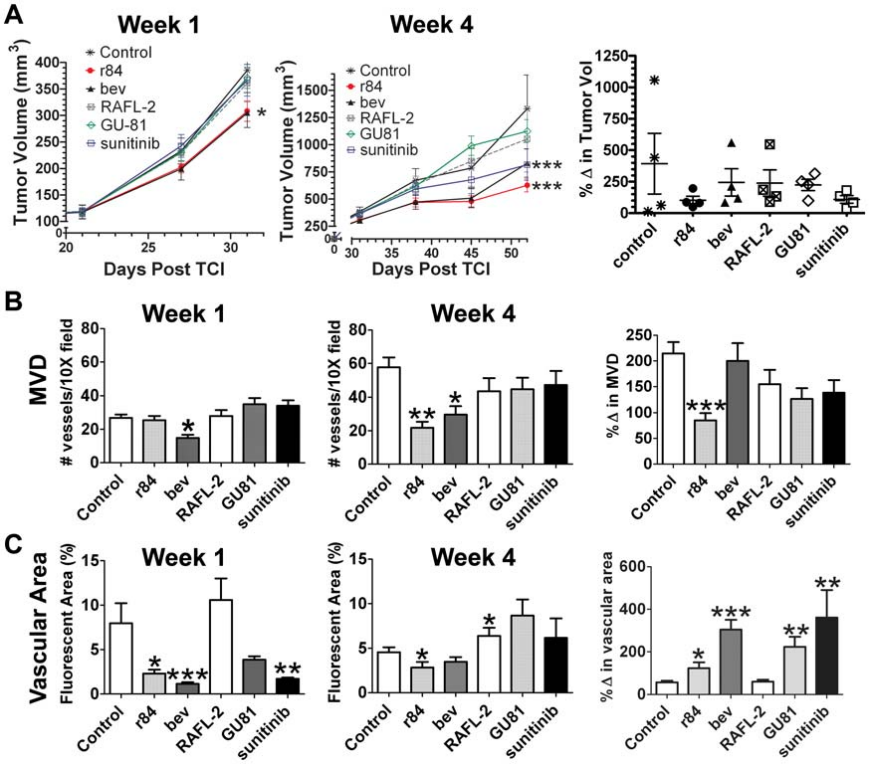
Anti-tumor and anti-vascular effects of VEGF pathway inhibition in MDA-MB-231 xenografts. MDA-MB-231 human breast cancer cells (5×10^6^) were injected into the mammary fat pad of SCID mice. Treatment with control IgG, bevacizumab (bev), r84, or RAFL-2 (250 µg twice weekly), GU81 (120 µg daily), sunitinib (200 µg daily) was initiated in established tumors (∼150 mm^3^) on day 24 post tumor cell injection (TCI) and continued for 1 (Week 1) or 4 (Week 4) weeks. (A) Mean tumor volume after 1 (n = 12/group) and 4 (n = 4/group) weeks of therapy is displayed. The mean percent change in tumor volume from 1 to 4 weeks of therapy is displayed as a scatter plot and was determined by dividing the tumor volume from individual mice at after 4 weeks of therapy by the mean tumor volume of the group after 1 week of therapy (n = 4/group/timepoint). (B–C) Tumor sections were analyzed by immunofluorescence using MECA-32, an endothelial cell marker for microvessel density (MVD, B) and vascular area (C). Data are displayed as mean±SEM and represents 5 images (Total magnification, 100X) per tumor and three tumors per group. Images were analyzed using Elements software. *p = 0.05, **p = 0.01, ***p<0.001.

Anti-VEGF therapy has been validated clinically in many types of cancer, including breast cancer [11], [33]. However, few studies have investigated whether differential blockade of the VEGF pathway results in differential effects on tumor growth and the tumor microenvironment in breast cancer. We studied the effect of selectively blocking the VEGF pathway using the agents listed in Table 1 in mice bearing established MDA-MB-231 human breast tumor xenografts. The effect of therapy with all six agents was evaluated after one and four weeks of drug exposure. After one week of therapy, only tumors from mice treated with r84 or bevacizumab were significantly smaller than control-treated tumors (Fig. 1A). After four weeks of therapy, selective blockade of VEGF binding to VEGFRs (r84; bevacizumab) and the RTKI (sunitinib) significantly limited tumor growth compared to control treatment (Fig. 1A). Tumors treated with agents that selectively block VEGFR2 (RAFL-2) or both receptors (GU81) did not control tumor growth compared to control IgG at the one and four week time points. Tumor volume increased an average of 393% from day 31 (week 1) to day 52 (week 4) post tumor cell injection (TCI) time in mice receiving a control IgG. Treatment with r84, bevacizumab, RAFL-2, GU81, and sunitinib resulted in mean tumor volume increases of 102, 244, 239, 224, and 109%, respectively. Each of these inhibitors block VEGFR2 activity (Fig. 1A). These results support the concept that selective inhibition of VEGFR2 is adequate to control the growth of human breast tumor xenografts.

**Table 1:** Anti-VEGF Agents^a^.

Agent	Class	Target	Target Species
r84	Human Ab	VEGF (blocks VEGFR2 only)	Mouse & Human
mcr84	Murine chimeric Ab	VEGF (blocks VEGFR2 only)	Mouse & Human
bevacizumab	Humanized Ab	VEGF (blocks VEGFR1 and VEGFR2)	Human
RAFL-2	Rat Ab	VEGFR2	Mouse
GU81	Peptoid	VEGFR1/2	Mouse & Human
sunitinib	Small molecule	VEGFR1/2, cKit, PDGFRβ	Mouse & Human

a Ab: antibody; VEGF: Vascular endothelial growth factor; VEGFR1: VEGF receptor 1; VEGFR2: VEGF receptor 2; PDGFRβ: Platelet-derived growth factor receptor β.

To determine the effect of anti-VEGF therapy on angiogenesis, we assessed microvessel density (number of vessels/100X field) and vascular area (% positive fluorescent area/100X field) at the one and four week time points (Fig. S1A). Percent change (Δ) in MVD or vascular area was defined as week 4 MVD or vascular area/mean week 1 MVD or vascular area. After one week of therapy, only bevacizumab-treated tumors had significantly fewer vessels (p<0.05) compared to control-treated tumors (Fig. 1B). However, overall vascular area was decreased in tumors from animals treated with bevacizumab, r84 and sunitinib (Fig. 1C); indicating vessel size was decreased after anti-VEGF therapy. After four weeks of therapy (Fig. 1B), only r84 and bevacizumab reduced microvessel density compared to control IgG (p<0.001). Interestingly, r84 prevented an increase in MVD from week 1 to week 4 of therapy, while MVD increased in all other treatment conditions (Fig. 1B; % Δ in MVD). However, tumors from all anti-VEGF therapies, except RAFL-2 had an increase in vascular area over the course of therapy (Fig. 1C; % Δ in vascular area). In comparing the anti-angiogenic activity of these agents, we found that selectively blocking VEGF from binding VEGFR2 (r84) was as or more effective than all other anti-VEGF strategies in decreasing MVD and vascular area in MDA-MB-231 orthotopic xenografts.

### VEGF Is an Important Cytokine for Immune Cell Infiltration in MDA-MB-231 Human Breast Cancer Xenografts

**Figure 2:**
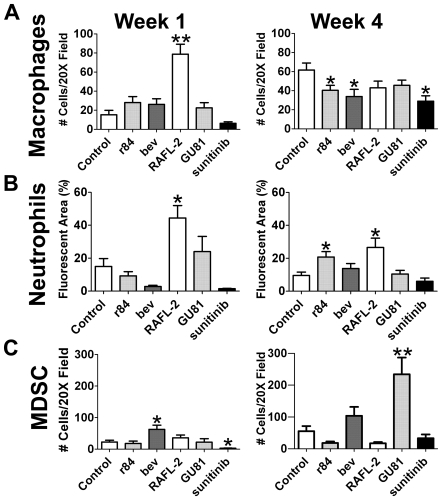
Inhibition of VEGF receptor activation utilizing different blocking strategies results in variations in immune cell infiltration in MDA-MB-231 human breast cancer xenografts. (A–C) Tumor sections were analyzed by immunofluorescence using F4/80, a macrophage marker, (A), and 7/4, a neutrophil marker (B). Tumor sections were evaluated by immunofluorescence for myeloid-derived suppressor cells (MDSCs, C) defined as the number of cells that express CD11b and Gr1 per 200X field. Data are displayed as mean±SEM and represents 5 images (Total magnification, 200X) per tumor and three tumors per group. Images were overlayed and analyzed using Elements software. *p = 0.05, **p = 0.01.

Previously, we have shown a reduction in macrophage and MDSC infiltration and an increase in neutrophil infiltration into xenografts following anti-VEGF therapy [14], [16], [22]. Surprisingly, after one week of therapy, selective blockade of VEGFR2 with RAFL-2 significantly induced macrophage infiltration into tumors compared with control treated animals (RAFL-2: 78.8±10.5 vs control: 15.4±4.5 cells/200X field; p<0.01; Fig. 2A; Fig. S1B). However, after four weeks of therapy, all anti-VEGF agents reduced macrophage infiltration (Fig. 2A). Neutrophil infiltration was examined using the anti-neutrophil antibody, 7/4. Acute selective inhibition of VEGFR2 (RAFL-2) induced neutrophil accumulation into tumors (Fig. 2B; Fig. S1C). While chronic inhibition of VEGFR2 activation by r84 and RAFL-2, but not other strategies, resulted in increased neutrophil accumulation in tumors (Fig. 2B). Next, we investigated the effect of anti-VEGF therapy on MDSC infiltration (CD11b^+^Gr1^+^ cells) into tumors. Bevacizumab treatment resulted in MDSC accumulation after 1 and 4 weeks of therapy, although the increase was only significant after 1 week (Fig. 2C; Fig. S1D). Sunitinib on the other hand reduced the number of MDSC cells at both time points although again this was only significant after one week of therapy (Fig. 2C). GU81, which binds both VEGFR1 and VEGFR2 had little effect on MDSC numbers after 1 week of therapy; however at the 4 week time point, there was a significant increase in MDSC infiltration compared to control-treated animals (Fig. 2C). r84 and RAFL-2 had no discernable effect on MDSC numbers after one week of therapy although each reduced MDSC infiltration after 4 weeks of treatment (Fig. 2C; Fig. S1D). How VEGF governs MDSC recruitment into tumors is unclear. Our data suggests that blockade of VEGFR1 and VEGFR2 (e.g., bevacizumab and GU81) can induce an increase in MDSC infiltration, while selective blockade of VEGFR2 limits MDSC accumulation in this xenograft model of breast cancer.

### Mouse Chimeric r84 Delays Tumor Growth and Improves the Immune Profile in Inflammatory 4T1 Breast Tumors

**Figure 3:**
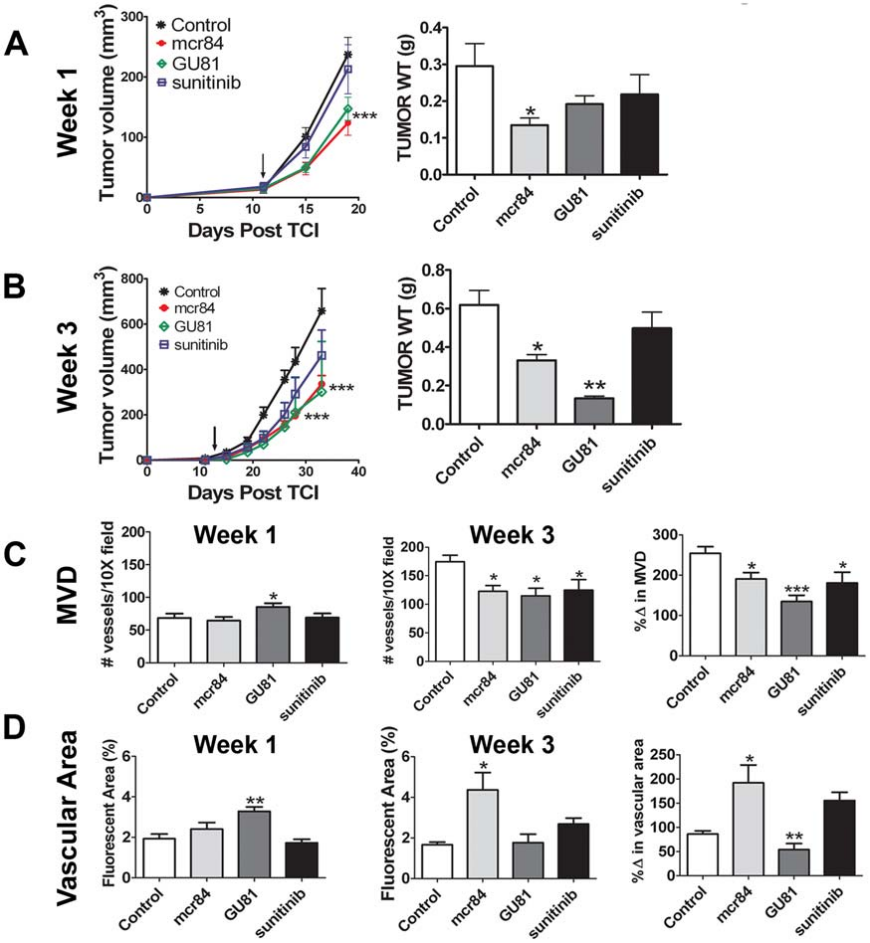
Anti-VEGF therapy delays tumor growth and reduces microvessel density in the inflammatory 4T1 breast cancer model. (A–B) 4T1 murine breast cancer cells (1×10^5^) were injected into the mammary fat pad of BALB/c mice. Treatment with 250 µg twice weekly of either control IgG or mcr84, 120 µg daily GU81 or 200 µg daily sunitinib was initiated in established tumors (∼15 mm^3^) on day 12 post tumor cell injection (TCI; arrow) and continued for 1 (A) or 3 (B) weeks (n = 4/group/timepoint). Tumor volumes were measured twice weekly and mean tumor volume +/− SEM is displayed. (C–D) Tumor sections were analyzed by immunofluorescence using MECA-32, an endothelial cell marker at the one and three week time points for microvessel density (MVD, C) and vascular area (D). Data are displayed as mean±SEM and represents 5 images (Total magnification, 100X) per tumor and three tumors per group. Images were analyzed using Elements software. *p = 0.05, **p = 0.01, ***p<0.001.

Growth of 4T1 tumors cells in the mammary fat pad of BALB/c mice is an inflammatory model of breast cancer in which immune cells comprise 40-50% of the overall tumor mass [34], [35]. To extend our previous observations, we performed similar experiments utilizing this immunocompetent model of breast cancer. By qRT-PCR, we demonstrate that 4T1 cells express VEGFR1 but not VEGFR2 *in vitro* (Table S1). For *in vivo* studies, mice with small but established tumors were treated for 1 or 3 weeks with a control IgG, a mouse chimeric version of r84 (mcr84), GU81, or sunitinib (Table 1). As seen in the MDA-MB-231 model, after one and three weeks of therapy, inhibition of mouse VEGF binding to VEGFR2 (mcr84) significantly reduced tumor growth compared to control IgG (Fig. 3A). Interestingly, GU81 controlled tumor growth after one and three weeks, while sunitinib had little effect on tumor volume or weight at either time point (Fig. 3A).

**Figure 4:**
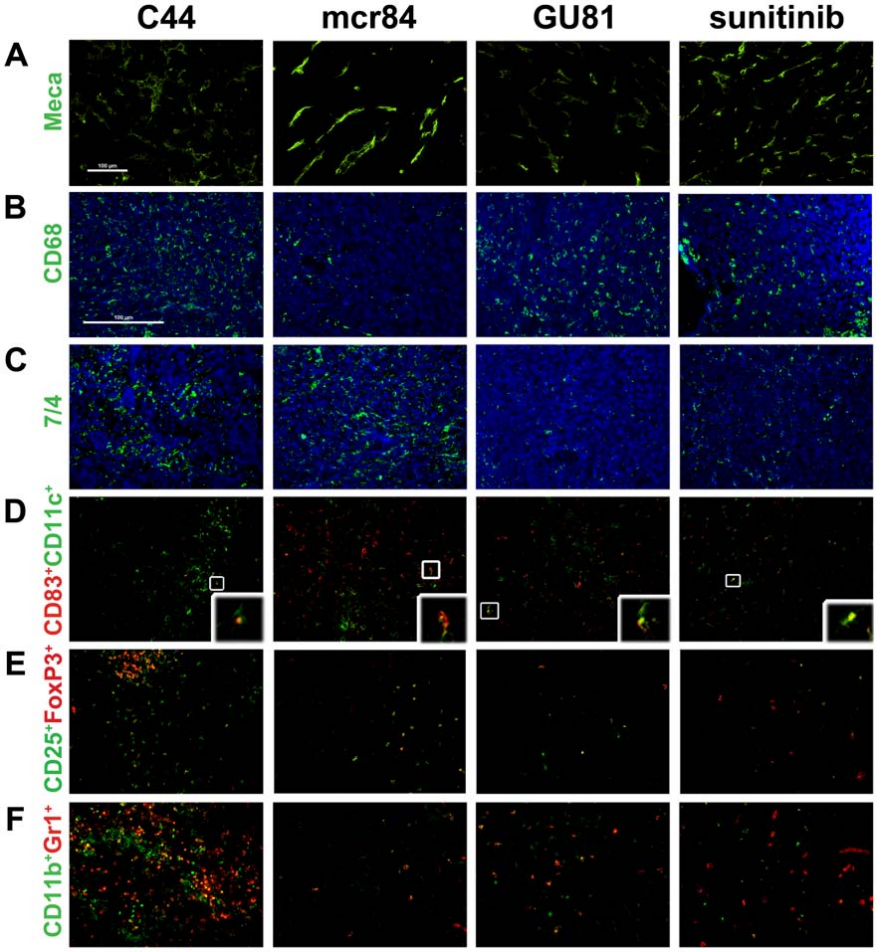
Representative immunofluorescence images of microvessel density and immune cell infiltration in the 4T1 model. Tumor sections from mice treated with the indicated anti-VEGF agent were analyzed by immunofluorescence using MECA-32, an endothelial cell marker (A), CD68, a macrophage marker (B), and 7/4, a neutrophil marker (C). Tumor sections were evaluated by immunofluorescence for mature dendritic cells by co-localization (box) of CD83 and CD11c (D); Treg by co-localization of CD25^+^and FoxP3^+^ cells (E); and myeloid-derived suppressor cells defined as the number of cells that express CD11b and Gr1 per 200X field (F). The inset on each picture in row D is a magnified view of co-localization of CD83 and CD11c. Representative pictures of control and anti-VEGF treated tumors are displayed. Total magnification, 200X; except for Meca-32 staining (100X), scale bar, 100 µm. Images were overlayed and analyzed using Elements software. Quantitation of signal intensity is shown in Figures 3 and 5.

Though mcr84 and GU81 limited tumor growth after one week of therapy, tumors from GU81-treated animals had increased MVD and vascular area (Fig. 3C and D, week 1). In contrast, after three weeks of therapy, tumors from all treatment groups had a reduction in MVD compared to control (Fig. 3C, week 3; Fig. 4A). Unpredictably, tumors from animals treated with mcr84 had increased vascular area compared to control-treated tumors (4.37%±0.85 vs 1.67%±0.13, respectively; p<0.05) after three weeks of therapy (Fig. 3D). However, all three agents prevented an increase in MVD from week 1 to week 3 of therapy (Fig. 3C; % Δ in MVD). These surprising vascular changes following mcr84 and GU81 therapy were validated using two additional endothelial cell markers, endomucin and CD31 (Fig. S2A–C).

**Figure 5:**
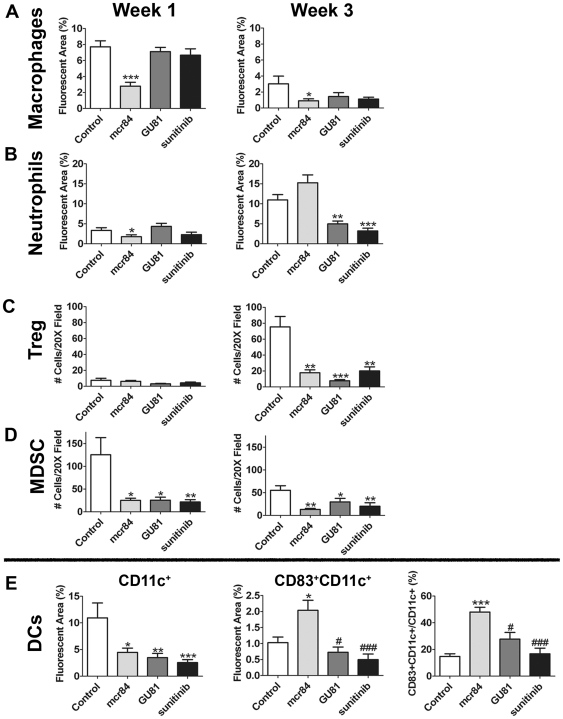
mcr84 reduces immune suppressor cells and increases mature dendritic cell infiltration in the inflammatory 4T1 breast cancer model. (A–B) Tumor sections were analyzed by immunofluorescence at the one and three week time points using CD68, a macrophage marker (A), and 7/4, a neutrophil marker (B). (C–E) Tumor sections were evaluated by immunofluorescence for (C) Tregs, colocalization of CD25^+^ and FoxP3^+^ (D) and MDSCs, colocalization of CD11b^+^ and Gr1^+^. Dendritic cells were characterized using CD11c^+^ (total DCs), mature dendritic cells, defined as colocalization of CD83 and CD11c and % of CD11c^+^ cells that were CD83^+^ (Week 1 only). Data are displayed as mean±SEM and represents 5 images (Total magnification, 200X) per tumor and three tumors per group. Images were overlayed and analyzed using Elements software. *p = 0.05, **p = 0.01, ***p<0.001 vs control; #p = 0.05, ###p<0.001 vs mcr84.

Similar to MDA-MB-231 tumors, inhibition of VEGF resulted in reduced macrophage infiltration (CD68^+^ cells). This was evident with mcr84 at both time points. GU81 and sunitinib also reduced macrophage numbers after three weeks of therapy although the changes did not reach statistical significance (Fig. 4B, Fig. 5A). We also found that neutrophil infiltration (7/4^+^ cells) was reduced significantly following chronic therapy with GU81 or sunitinib (Fig. 4C, Fig. 5B).

Tregs were identified by the co-expression of CD25 and FoxP3. Though we did not see any significant changes in Treg infiltration after one week of anti-VEGF therapy (Fig. 5C), chronic anti-VEGF therapy inhibited the infiltration of Treg into tumors compared to control IgG (Fig. 4E, Fig. 5C). In the MDA-MB-231 model, we found a significant increase in MDSCs in tumors treated with chronic GU81 (Fig. 2C). However, in the 4T1 breast cancer model, we found a significant reduction in MDSC infiltration in all anti-VEGF groups at both time points (Fig. 4F, Fig. 5D).

Previously, we identified an increase in CD83^+^CD11c^+^ mature dendritic cells in MDA-MB-231 tumors treated with r84 [14]. We found a similar effect in the 4T1 model, whereby there was an overall decrease in CD11c^+^ cells in all treatment groups after one week of therapy (Fig. 5E). However, when we looked at the number of CD83^+^CD11c^+^ mature dendritic cells (Fig. 4D, Fig. 5E), tumors from animals treated with mcr84 had a significant increase in this population of cells compared to all other treatment groups. Furthermore, when we specifically analyzed the CD11c^+^ population of cells, we found that approximately 48% of dendritic cells within mcr84 tumors expressed CD83, whereas only 14.8, 27.8 and 16.8% of dendritic cells in control, GU81 or sunitinib, respectively, expressed CD83 (Fig. 5E; p<0.001).

### Effect of Anti-VEGF Therapy on Immune Cell Infiltration in the Transgenic MMTV-PyMT Breast Tumor Model

The MMTV-PyMT transgenic mouse expresses the polyomavirus middle T antigen driven by the MMTV-LTR promoter [36]. Polyomavirus middle T oncogene expression results in the generation of multifocal mammary carcinomas in 100% of female mice. We treated 52 day old transgenic MMTV-PyMT females with control IgG, mcr84, GU81 or sunitinib for four weeks (Table 1). Similar to the MDA-MB-231 model, we found that mcr84 controlled tumor growth after four weeks of therapy compared to control-treated tumors (Fig. S3A). Tumors from mcr84-treated animals had a decrease in vascular area and macrophage infiltration (CD11b^+^Gr1^−^ cells) compared to control-treated tumors (Fig. S3B, C) and an increase in neutrophils (CD11b^−^Gr1^+^ cells; Fig. S3D). Additionally, mcr84-treated tumors also had a significant reduction in MDSC and Treg infiltration compared to control, GU81, and sunitinib treated tumors (Fig S3E, F).

With these studies, we have extended our previous observations [14] into an immunocompetent model system and have further validated that VEGF is an important cytokine that regulates immune cell trafficking into breast tumors.

### Tumor Cytokine Profile Changes Induced by Anti-VEGF Therapy

**Figure 6:**
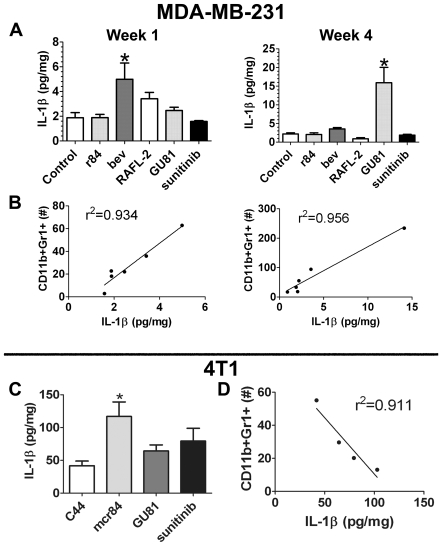
Changes in intra-tumoral IL-1β levels following anti-VEGF therapy correlate with intra-tumoral MDSCs. (A) MDA-MB-231 intra-tumoral IL-1β levels after one and four weeks of anti-VEGF therapy were determined by electrochemiluminescence. (B) By linear regression analysis, changes in MDA-MB-231 intra-tumoral IL-1β levels following anti-VEGF therapy positively correlate with intra-tumoral CD11b^+^Gr1^+^ (MDSCs) after one and four weeks of therapy. Each dot represents the mean IL-1β and MDSC for each treatment group. (C) 4T1 intra-tumoral IL-1β levels after three weeks of anti-VEGF therapy were determined by electrochemiluminescence. (D) By linear regression analysis, changes in 4T1 intra-tumoral IL-1β levels following anti-VEGF therapy negatively correlate with intra-tumoral CD11b^+^Gr1^+^ (MDSCs) after three weeks of therapy. Each dot represents the mean IL-1β and MDSC for each treatment group. *p = 0.05

To address some of the differences we found in immune cell infiltration with anti-VEGF therapy, we analyzed intra-tumoral cytokine levels in tumor lysates from the various treatment groups in the MDA-MB-231 and 4T1 models (Tables S2 & S3). MDSC accumulation is driven by many factors, including VEGF and IL-1β [17], [37], [38]. Given that there were differences in MDSC infiltration following different anti-VEGF therapies, we hypothesized that this may be due to aberrations in intra-tumoral IL-1β levels. In the MDA-MB-231 model, we found that inhibition of both VEGFR1 and VEGFR2 activation either via blocking ligand binding (bevacizumab) or receptor activation (GU81) resulted in significant increases in MDSC infiltration (Fig. 2C). In this model, intra-tumoral IL-1β levels were increased significantly following one week of bevacizumab therapy and four weeks of GU81 therapy (Table 1; Fig. 6A). By linear regression analysis, we found that changes in IL-1β levels as a result of anti-VEGF therapy were highly correlative with changes in MDSC infiltration at the one and four week time points (Fig. 6B).

In the 4T1 model tumors from mcr84-treated animals had increased levels of IL-1β after three weeks of therapy (Fig. 6C). Furthermore, following one week of therapy we found a trend in which increases in IL-1β correlated positively with MDSC infiltration (Table 2). However, after chronic anti-VEGF therapy (three week time point), increases in intra-tumoral IL-1β levels correlated negatively with changes in intra-tumoral MDSCs (Fig. 6D; Table 2). These results suggest that IL-1β has a bi-modal effect on MDSC migration such that IL-1β concentrations ≤5 pg/mg or ≥50 pg/mg result in reduced recruitment of MDSCs. We also evaluated intra-tumoral levels of IL-6, a downstream effector of IL-1β previously shown to be important for MDSC infiltration [37], and found that levels of this cytokine did not correlate with MDSC number after 3 weeks of therapy. This indicates that IL-6 is not the downstream mediator of IL-1β-mediated MDSC infiltration in the 4T1 breast cancer model, and suggests that another downstream target of IL-1β may modulate MDSC infiltration in this model.

**Table 2:** Changes in 4T1 intra-tumoral cytokine levels with anti-VEGF therapy correlate with changes in immune cell infiltration^a^.

Week 1							Week 3						3
r^2^	IL-1β	IL-6	CXCL1	TNF-α	IFN-γ	IL-2	r^2^	IL-1β	IL-6	CXCL1	TNF-α	IFN-γ	IL-2
IL-6	**0.825**						IL-6	**0.818**					
CXCL1	**0.834**	**0.952**					CXCL1	**0.945**					
TNF-α	**0.967**	**0.804**	**0.882**				TNF-α	*0.668*	*0.964*				
IFN-γ	**0.959**	**0.934**	**0.953**	**0.961**			IFN-γ						
IL-2	**0.978**	**0.899**	**0.858**	**0.916**	**0.966**		IL-2						
IL-4	**0.901**	0.75	0.647	0.767	**0.819**	**0.936**	IL-4						
IL-10	**0.884**	**0.865**	0.743	0.763	**0.866**	**0.955**	IL-10						
MDSC	0.741	**0.937**	**0.793**		**0.818**	**0.858**	MDSC	*0.911*	0.418	*0.986*			
Treg	**0.964**	0.671	0.725	**0.956**	**0.878**	**0.891**	Treg				0.138	**0.831**	0.305
**Vascular area**							**Vascular Area**	**0.845**	**0.894**	0.642			

a Data are displayed as r^2^ as determined by Pearson correlation test; p<0.075 for all bolded values. Values in *italics* indicate significant negative correlation; values in **bold** indicate a significant positive correlation. For example, as graphically displayed in Figure 5D, as IL-1β levels increase following three weeks of anti-VEGF therapy (week 3, column1) MDSCs infiltration decreases (row 8; r^2^ = 0.911)

**Figure 7:**
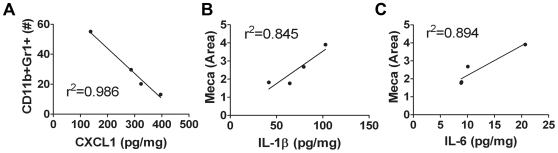
CXCL1, IL-1β, and IL-6 are differentially related to MDSC infiltration and vascular area in 4T1 tumors following anti-VEGF therapy. (A–C) Intra-tumoral IL-1β, CXCL1 and IL-6 were determined by electrochemiluminescence and ELISA. (A) Changes in intra-tumoral CXCL1 levels following anti-VEGF therapy negatively correlate with intra-tumoral CD11b^+^Gr1^+^ (MDSCs) after three weeks of therapy. (B–C) Changes in intra-tumoral IL-1β (B) and IL-6 (C) positively correlate with vascular area after chronic anti-VEGF therapy. Each dot represents the mean cytokine level for each treatment group. N = 4/grp, assayed in duplicate.

CXCL1 (KC, GRO) is expressed by macrophages, neutrophils, endothelial cells and has neutrophil chemoattractant activity [39]. IL-1β can induce CXCL1 expression via a different mechanism than IL-6 [40]. In 4T1 tumors, we found that changes in CXCL1 levels were correlative with changes in IL-1β after one and three weeks of anti-VEGF therapy (Table 2, Table S3). Furthermore, changes in CXCL1 levels with anti-VEGF therapy negatively correlate with changes in MDSC infiltration at both time points (Fig. 7A; Table 2), suggesting an alternative mechanism for MDSC recruitment in the presence of anti-VEGF therapy and increased levels of IL-1β.

Previously, we have shown that mice treated with r84 have no detectable level of free VEGF in serum [14]. Therefore, we sought to investigate an alternative mechanism for the increased vascular area seen in 4T1 tumor-bearing animals treated chronically with mcr84 (Fig. 3D). Though VEGF is the primary stimulant for tumor angiogenesis, IL-1β can also stimulate *in vitro* endothelial cell migration and proliferation [41] and angiogenesis in mouse models of cancer via up regulation of VEGFR2 [42], [43]. mcr84-treated tumors had a three-fold increased expression of VEGFR2 compared to control-treated tumors (Fig. S2C). Furthermore, we found by linear regression analysis, that increases in vascular area seen with anti-VEGF therapy correlated with changes in IL-1β and IL-6 (Fig. 7B & C; Table 2), suggesting an alternative pathway for angiogenesis in the presence of anti-VEGF therapy.

The development, recruitment and activation of regulatory T cells (Treg) in the tumor microenvironment is not completely understood. Though TGF-β can induce peripheral Treg, it is not required for the generation of a thymic-derived subset of these cells. Recently, IL-2, IFN-γ and TNF-α have been implicated in Treg generation [30], [31], [32]. In this study we found that only IFN-γ levels correlate with Treg infiltration during both acute and chronic therapy, indicating IFN-γ may direct the development or migration of Treg in the face of anti-VEGF therapy (Table 2).

### Identification of Potential Biomarkers of Response

**Figure 8:**
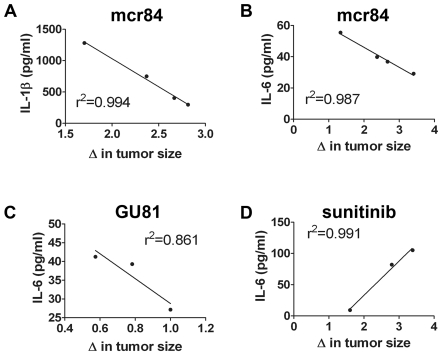
Identification of potential biomarkers of response. Serum levels of IL-1β and IL-6 were determined by electrochemiluminescence and ELISA, respectively from animals treated with anti-VEGF therapy bearing 4T1 tumors. Change (Δ) in tumor size was calculated as week three tumor weight/mean week one tumor weight for each individual therapy group. (A-C) By linear regression analysis, changes in serum levels of IL-1β (A) and IL-6 (B,C) negatively correlate with Δ in tumor size for animals treated with mcr84 and GU81. (D) Changes in IL-6 levels with sunitinib therapy positively correlate with Δ in tumor size.

Given the changes we observed in inflammatory cytokines in response to various anti-angiogenesis strategies, we sought to investigate whether changes in serum levels of these cytokines would correlate with tumor progression. High levels of serum IL-1β and IL-6 levels correlated with delayed tumor progression in animals bearing 4T1 tumors treated with mcr84 and GU81, whereas low levels of these cytokines corresponded to tumor progression (Fig. 8A–C). However, low levels of IL-6 in the sera of sunitinib-treated animals correlated with tumor progression (Fig. 8D). These findings highlight the importance of the inflammatory cytokine profile in tumor progression and identify possible biomarkers of response to r84 or other anti-VEGF agents in breast cancer.

## Discussion

In this report, we provide data that demonstrate the effectiveness of anti-VEGF therapy as a modulator of immune cell infiltration, and intra-tumoral and serum cytokine levels in multiple preclinical models of breast cancer. It is becoming increasing clear that the effect of anti-VEGF agents extends beyond the inhibition of angiogenesis, as many immune cells express VEGFRs, including macrophages, neutrophils, MDSCs, DCs and T-cells [6], [17], [26], [44], [45]. We and others have shown a reduction in macrophages in tumors from animals treated with anti-VEGF therapy [14], [16], [23]. In preclinical models of colon cancer, sunitinib treatment reduced the accumulation of MDSCs and plasmacytoid dendritic cells in tumors compared to control treatment [20]. In metastatic renal cell carcinoma patients, sunitinib therapy reduced the level of circulating MDSCs and reduction in MDSCs in response to sunitinib therapy correlated with an increase in T-cell IFN-γ production [18]. These studies suggest that sunitinib or other anti-VEGF therapies function as modulators of antitumor immunity. Here, we demonstrate that inhibition of VEGF binding to VEGFR2 with r84 is more effective than other anti-VEGF strategies in controlling breast tumor growth and the infiltration of immune suppressor cells.

Using the MDA-MB-231 human breast cancer model, we demonstrate that inhibition of VEGF binding to VEGFRs with bevacizumab or r84 effectively controls tumor growth (Fig. 1A). Furthermore, only selective inhibition of VEGF binding to VEGFR2 with r84 is able to prevent an increase in MVD from week 1 to week 4 of therapy (Fig. 1B). Interestingly, when these experiments were repeated using the immunocompetent 4T1 inflammatory breast cancer model, mcr84 and the VEGFR1 & VEGFR2 binding peptoid, GU81, were able to control tumor growth (Fig. 3A and B). Though mcr84 was able to reduce MVD as seen in the MDA-MB-231 model, 4T1 tumors from animals treated with chronic mcr84 had an increase in vascular area compared to control (Fig. 3C and D). In an effort to explain this increase in vascular area, we evaluated intra-tumoral cytokine levels in tumors from all treatment groups at the one and three week time points (Table S3). Though hypoxia and VEGF are the main angiogenic stimuli, other cytokines, including IL-1β, IL-6 and TNF-α can induce angiogenesis [42], [43], [46], [47]. For example, in cardiac myocytes IL-1β increases VEGFR2 expression [43]. In mcr84-treated tumors, we found increased levels of IL-1β and VEGFR2 compared to control-treated tumors (Fig. 6C, Fig. S2C). By linear regression analysis, increases in intra-tumoral IL-1β following anti-VEGF therapy correlated with increased vascular area (Fig. 7B). VEGFR2 is the main receptor responsible for VEGF-induced angiogenesis and is activated by VEGF-A, C, or D [48], [49]. It is plausible that VEGF-C/D may bind to increased levels of VEGFR2, resulting in increased VEGFR2 phosphorylation and signaling. Increased VEGFR2 signaling can then promote the increased vascular area observed in mcr84- treated tumors.

The effects of anti-VEGF therapy extend beyond its effects on tumor blood vessels. In both the MDA-MB-231 and 4T1 models, chronic anti-VEGF therapy reduced macrophage infiltration in all treatment groups. In non-tumor bearing animals, monocyte and macrophage migration is driven in part by placental growth factor (PlGF), VEGF and VEGFR1 [50]. However, we have shown previously that VEGFR2 is expressed on macrophages from tumor-bearing animals, and is the dominant receptor driving VEGF-induced macrophage chemotaxis in tumor-bearing animals [14], [16]. Therefore, the reduction in macrophage migration seen following anti-VEGF therapy is likely due to the inhibition of VEGFR2 signaling (Fig. 2A; Fig. 5A). Interestingly, the inhibition of both VEGFR1 and VEGFR2 activation with bevacizumab, GU81, or sunitinib did not reduce macrophage infiltration better than agents that inhibited the activation of VEGFR2 alone (r84 or RAFL-2).

Neutrophils are often described as ‘first responders’ and have been shown to be capable of mediating the angiogenic switch in engineered animal models of cancer [23], [51]. The mechanism underlying the increase in 7/4^+^ cells after anti-VEGF therapy is unclear. VEGF can stimulate neutrophil migration *in vitro* via VEGFR1 activation [19]. Furthermore, these cells were shown to express VEGFR1 and VEGFR2 by RT-PCR, suggesting that even though VEGFR2 is present, VEGFR1 is the primary receptor mediating VEGF-induced migration of these cells. In support of this, we have previously shown an increase in neutrophil infiltration in r84-treated tumors [14]. In this study, we have further characterized the effect of anti-VEGF therapy on neutrophil infiltration utilizing an immunocompetent model of breast cancer. In the 4T1 model, tumors from animals treated with agents that block VEGFR1 activation (GU81 and sunitinib) had reduced neutrophil infiltration compared to control-treated tumors. In contrast, tumors from animals treated with mcr84, where VEGFR1 signaling was intact, had an increase in neutrophil infiltration (Fig. 5B), suggesting that VEGFR1 is the dominant receptor involved in VEGF-mediated neutrophil migration in tumor-bearing animals.

VEGF is a key mediator in the development and maturation of dendritic cells [17]. Activation of VEGFR1 on dendritic cells inhibits hematopoetic stem cell differentiation along the dendritic cell lineage, whereas VEGFR2 is important for dendritic cell maturation [17]. Previously, using the MDA-MB-231 model, we found an increase in mature dendritic cells in animals treated with r84, but not bevacizumab [14]. In this study, we demonstrate that this effect is seen after only one week of therapy, as inhibition of VEGF binding to VEGFR2 with mcr84 reduced the number of total dendritic cells, while increasing the mature fraction of these cells (Fig. 5E). Though the antigen presenting ability of these cells is not known, in human breast cancer specimens, increased CD83+ dendritic cells is associated with an improved prognosis [52].

The role of VEGF in myeloid-derived suppressor cells (MSDC) differentiation and migration has been characterized in recent years [25], [53]. In mouse models and patients with cancer, these immunosuppressive cells are increased in the blood, spleen and tumors [18], [20], [53]. Many factors induce MDSC expansion and activation, including VEGF, IL-1β, and IL-6, making these attractive targets for MDSC inhibition. In this study, we reveal an interesting connection between these cytokines in mediating MDSC infiltration into tumors. In MDA-MB-231 tumors, treatment with anti-VEGF agents that block both VEGFR1 and VEGFR2 (bevacizumab and GU81), but not other receptor tyrosine kinases resulted in increased intra-tumoral IL-1β levels and MDSC accumulation (Fig. 2C, Fig. 6A). It is interesting to note that sunitinib, which also blocks both VEGFR1 and VEGFR2 signaling, does not result in increased IL-1β and MDSC accumulation. This is likely due to the fact that sunitinib also blocks PDGFRβ, GSF-1R, Flt-3, and cKit, any of which may be important for increased expression of IL-1β. Furthermore, changes in IL-1β levels in response to anti-VEGF therapy were highly correlative with changes in MDSC infiltration at the one and four week time-points (Fig. 6B), indicating that IL-1β is a key cytokine mediating the infiltration of MDSCs following anti-VEGF therapy. Interestingly, in the 4T1 immunocompetent model of breast cancer, changes in intra-tumoral IL-1β levels correlate negatively with changes in MDSC infiltration after three weeks of therapy (Fig. 6D, Table 2), where increases in IL-1β following treatment with mcr84 were associated with reduced MDSC infiltration. These findings indicate a possible bimodal role of IL-1β in MDSC infiltration, where a low level of IL-1β induces MDSC infiltration and increased levels following anti-VEGF therapy inhibit MDSC infiltration.

MDSCs do not express receptors for IL-1β; however, they do express receptors for IL-6, which is capable of inducing MDSC infiltration in the absence of IL-1β signaling [38]. Therefore, we investigated other cytokines that may be involved in regulating immune cell recruitment. Similar to IL-1β, tumors from animals treated chronically with mcr84 had increased IL-6 and CXCL1 levels. However, only changes in CXCL1, not IL-6, correlated negatively with changes in MDSC infiltration, as seen with IL-1β (Fig. 7A, Table 2). Therefore, we propose that CXCL1 is an inhibitor of MDSC infiltration subsequent to markedly increased IL-1β levels following anti-VEGF therapy.

Treg are immune suppressor cells that maintain peripheral tolerance [28]. Like MDSCs, these cells are increased in the blood and tumors of cancer patients and mouse models of cancer [28]. The generation of Treg is a complicated process that involves many cytokines, such as IL-2, TGF-β, IFN-γ and TNF-α [30], [31], [32]. Though acute anti-VEGF therapy did not affect Treg infiltration, we found reduced levels of Treg after chronic anti-VEGF therapy in all treatment groups (Fig. 4E; Fig. 5C). Furthermore, only changes in intra-tumoral IFN-γ levels correlated with changes in Treg infiltration, further confirming its importance in Treg infiltration/generation (Table 2).

Finally, having demonstrated changes in intra-tumoral cytokine levels and immune cell infiltration with anti-VEGF therapy, we looked at serum levels of IL-β and IL-6, as potential biomarkers of response to anti-VEGF therapy. For animals treated with mcr84 or GU81, we found that decreases in serum levels of IL-1β and IL-6 were highly correlative with changes in tumor size in the presence of anti-VEGF therapy (Fig. 8A–C). Interestingly, increases in serum IL-6 levels in sunitinib-treated animals correlated with increases in tumor size, suggesting different mechanisms for the cytokine aberrations seen between selective versus broad spectrum anti-VEGF strategies (Fig. 8D).

In conclusion, we have demonstrated differences in the ability of anti-VEGF therapy to affect tumor vasculature and modulate immune cell infiltration, intra-tumoral and serum cytokine levels depending on the mechanism of VEGF inhibition. We have demonstrated that selective inhibition of VEGF binding to VEGFR2 with r84 is effective at controlling tumor growth, inhibiting the infiltration of suppressive immune cells (MDSC, Treg, macrophages) while increasing the mature fraction of dendritic cell infiltrates. Furthermore, we have identified two possible biomarkers (IL-1β and IL-6) for assessing the efficacy of anti-VEGF therapy in breast cancer patients.

## Materials and Methods

### Cell Lines, Culture Conditions

The human breast carcinoma cell line MDA-MB-231 and murine breast carcinoma cell line 4T1 were obtained from ATCC (Manassas, VA). Cells were maintained at 37°C in a mixture of 5% CO_2_ and 95% air in Dulbecco's minimal essential medium (DMEM, Invitrogen; Carlsbad, CA; MDA-MB-231 cells) or RPMI 1640 (Invitrogen, Carlsbad, CA; 4T1 cells) supplemented with 10% fetal calf serum (Gemini Bio-Products, Woodland, CA). Cell lines were confirmed to be pathogen free and human cell lines were genotyped to confirm origin prior to implantation into animals.

### Animal Tumor Models

6–8 week old female NOD/SCID mice or BALB/c were purchased from an on-campus supplier. The MMTV-PyMT transgenic mice in the FVB background (The Jackson Laboratory, Bar Harbor, ME) express the polyomavirus middle T antigen driven by the MMTV-LTR promoter [36]. Polyomavirus middle T oncogene expression results in the generation of multifocal mammary carcinomas in 100% of female mice, followed by progression to pulmonary metastases in the vast majority of animals. Only female transgenic mice were used in these experiments and were obtained by breeding transgenic male mice with *wild-type* FVB female mice. Progeny were monitored for transgene expression by PCR analysis. Animals were housed in a pathogen free facility and all animal studies were performed on a protocol approved by the IACUC at the University of Texas Southwestern Medical Center. 5×10^6^ MDA-MB-231 or 1×10^5^ 4T1 cells were injected into the mammary fat pad (MFP) of SCID or BALB/c mice, respectively using previously described techniques [22], [54]. Caliper measurements were performed twice weekly and tumor volume was calculated as D×d^2^×0.52, where D is the long diameter and d is the perpendicular short diameter.

### Anti-VEGF Therapy (Table 1)

Bevacizumab (Avastin®, Genentech, South San Francisco, CA) was purchased from the clinical pharmacy at UT-Southwestern. r84 and mouse chimeric r84 (mcr84) were provided by Peregrine Pharmaceuticals, Inc (Tustin, CA). The production and full characterization of r84, a human IgG1 specific for VEGF-A will be described in detail in a forth-coming manuscript (Sullivan et al). The hybridoma producing RAFL-2, a rat IgG specific for VEGFR2 was obtained from Dr. Philip Thorpe and was produced and purified in our laboratory as described [55]. GU81, a peptoid that binds and specifically inhibits VEGFR1 and VEGFR2 was produced as described previously [56], [57], [58], [59]. Sunitinib, a receptor tyrosine kinase inhibitor which inhibits VEGFR1, VEGFR2, PDGFRβ, c-kit, Flt-3, and c-Ret was purchased from LC laboratories (Woburn, MA).

Therapy was initiated on day 26 post tumor cell injection for MDA-MB-231 experiments or day 12 for 4T1 experiments, when tumor volume reached approximately 150 mm^3^ or 15 mm^3^, respectively. Therapy in the MMTV-PyMT model was initiated when the mice reached 8 weeks of age. Animals were randomized to receive intraperitoneal injection of IgG control, r84, bevacizumab, or RAFL-2 (250 µg of the designated IgG) twice weekly (Tuesday & Friday), GU81 (120 µg daily by intraperitoneal osmotic pump; Alzet, Cupertino, CA), or sunitinib (200 µg/day by oral gavage). Animals were sacrificed at various time points post initiation of therapy: 1 week (MDA-MB-231 (n = 4/group) and 4T1 models (n = 6/group)), 3 weeks (4T1 model, n = 4/group) or 4 weeks (MDA-MB-231 and MMTV-PyMT models; n = 4/group). Tumor weights were determined at the time of sacrifice and necropsy.

### PCR

RNA was prepared using TRIzol (Invitrogen) according to the manufacturer's instructions. The quality of RNA was evaluated using spectrophotometry. The cDNA used for subsequent for PCR was made using iScript (Bio-Rad Laboratories, Hercules, CA) and Choice DNA Taq polymerase (Denville Scientific, Metuchen, NJ) was used for subsequent PCR reactions. The expression of *VEGFR1* (Mm00438980_m1) *and VEGFR2* (Mm00440099_m1) was analyzed by quantitative real-time RT-PCR using an assay on demand (Mm00440111_m1) from Applied Biosystems. *GAPDH* (Applied Biosystems assay-on-demand) was used as an internal reference gene to normalize input cDNA. Quantitative real-time RT-PCR was performed in a reaction volume of 20 µl including 1 µl of cDNA, and each reaction was performed in triplicate. We used the comparative Ct method to compute relative expression values [60]. RNA isolated from murine brain endothelial cell line (bEnd.3) was used a positive control.

### Immunohistochemistry

Tissue was snap frozen in liquid nitrogen, embedded in OCT media, and sectioned. Sections were fixed in acetone, briefly air-dried and blocked with 20% Aquablock (East Coast Biologics, North Berwick, ME) for 30–60 minutes. Primary antibodies were used at a final concentration of 5–10 µg/ml and include: rat anti-mouse endothelial cell (MECA-32, Developmental Studies Hybridoma Bank, University of Iowa), rabbit anti-mouse CD31 (abcam, Cambridge, MA), rat anti-endomucin (sc-69495, Santa Cruz Biotechnology, Santa Cruz, CA), goat anti-F4/80 (sc-26642, Santa Cruz Biotechnology, Santa Cruz, CA), rat anti-CD68 (AbD Serotec, Raleigh, NC), rat anti-neutrophil, 7/4 (MCA 7716, AbD Serotec, Raleigh, NC), and rat anti-CD83 (Michel-19, BioLegend, San Diego, CA). Primarily conjugated antibodies include PE-labeled Gr1 (RB6-8C5), FITC-labeled CD11b (M1/70), Alexa Fluor® 488-labeled CD11c (N418) from Biolegend (San Diego, CA), Alexa Fluor® 488-labeled CD25 and Phycoerythrin-labeled FoxP3 from eBioscience (San Diego, CA). Primary antibody was incubated on sections for one hour at room temperature or overnight at 4°C. Negative controls were performed by omitting the primary antibody. Following washes, the appropriate fluorophore conjugated secondary antibody was added (Jackson Immunoresearch, West Grove, PA). Fluorescent slides were cover-slipped using Prolong with DAPI (Invitrogen, Carlsbad, CA). Sections were examined on a Nikon E600 microscope and images captured with Photometrics Coolsnap HQ camera using Elements Software. Fluorescent images were captured under identical conditions (exposure time, high and low limits, and scaling). Images were thresholded to exclude background signal from secondary antibody alone.

### ELISA/Electrochemiluminescence

Tumor lysates were made from orthotopic tumors by mincing the tumor in lysis buffer. Protein content was assayed using BCA assay (Pierce, Rockford, IL). Mouse total and active MMP-9, serum IL-1β and IL-6 Quantikine Immunoassays were performed according to manufacturer's instructions (R&D Systems, Minneapolis, MN). Active TGFβ levels were assessed using Promega TGFβ ELISA kit according to the manufacturer's instructions (Promega, Madison, WI). Electrochemiluminescence assays were performed on biological triplicate samples using capture antibody precoated 96-well multispot plates from Meso Scale Discovery (MSD; Gaithersburg, MD). 75 µg–100 µg of total protein was added to each well and incubated with shaking for 4 h at room temperature. Specific protein levels were quantitated by adding 25 µl of 1 µg/ml specific detection antibody labeled with MSD SULFO-TAG reagent to each well and incubated with shaking for 1 h at room temperature. The plate was then washed three times with PBS/0.05% Tween 20 and 150 µl of 2x read buffer was added to each well. Plates were immediately read using the SECTOR Imager 6000, and data were quantitated using Discovery Workbench and SOFTmax PRO 4.0 software.

### Statistics

Data were analyzed using GraphPad software (GraphPad Prism version 4.00 for Windows; GraphPad Software, San Diego, CA, www.graphpad.com). Results are expressed as mean±SEM. Spearman rank correlations were used to assess associations between immune parameters and cytokine levels. Data was analyzed by t-test or ANOVA and results are considered significant at p<0.05.

## Supporting Information

Figure S1 Representative images of immunohistochemistry staining for microvessel density and immune cell infiltration in MDA-MB-231 model. Tumor sections were analyzed by immunofluorescence using MECA-32, an endothelial cell marker (A), F4/80, a macrophage marker (B), and 7/4, a neutrophil marker (C). Tumor sections were evaluated by immunofluorescence for myeloid-derived suppressor cells defined as the number of cells that express CD11b and Gr1 per 200X field (D). Representative pictures of control (left column) and anti-VEGF treated tumors that had an increase (middle column) and a decrease (right column) in the indicated parameter. Total magnification, 200X; except for Meca-32 staining (100X). Images were overlayed using Elements software.
(6.43 MB PSD)

Figure S2 Effect of anti-VEGF therapy on vascular parameters in 4T1 tumors. Mice bearing established orthotopic 4T1 tumors were treated for 3 weeks with control IgG (C44) mcr84 (250 µg ip 2x/week), GU81 (120 µg/day via osmotic pump), or sunitinib (200 µg/day) (n = 4/group). Tumor sections (n = 4) were stained for CD31 (A), endomucin (B) or VEGFR2 (C) by immunofluorescence. Data are displayed as mean±SEM and represents 5 images per tumor and three tumors per group. Total magnification 100X. The mean fluorescent area was determined with Elements software. *, p<0.05; **, p<0.01 vs control by ANOVA.
(0.38 MB TIF)

Figure S3 Effect of anti-VEGF therapy on immune cell infiltration in the transgenic MMTV-PyMT breast tumor model. (A) Treatment with 250 µg twice weekly of either control IgG or mcr84, 120 µg daily GU81 or 200 µg daily sunitinib was initiated when transgenic females were 52 days old (arrow) and continued for 4 weeks (n = 5/group). Tumor volumes were measured twice weekly and mean tumor volume +/− SEM is displayed. (B–D) Tumor sections were analyzed by immunofluorescence using MECA-32, an endothelial cell marker (B), macrophages (CD11b+Gr1- cells) (C) and neutrophils (CD11b-Gr1+) (D). (E–F) Tumor sections were evaluated by immunofluorescence for (E) MDSCs, co-localization of CD11b+ and Gr1+ (F) Tregs, co-localization of CD25+and FoxP3+. Data are displayed as mean±SEM and represents 5 images per tumor and three tumors per group. Total magnification, 200X, except for Meca-32 staining (100X). Images were overlayed and using Elements software. *p = 0.05, **p = 0.01, ***p<0.001, ##p = 0.01 vs mcr84.
(3.41 MB TIF)

Table S1 4T1 cells express VEGFR1 but not VEGFR2. RNA isolated from murine (bEnd.3) endothelial cells and 4T1 cells was used for qRT-PCR analysis of VEGFR1 and VEGFR2 and normalized to GAPDH. The mean Ct (cycle threshold) value for each target is displayed.
(0.03 MB DOC)

Table S2 Anti-VEGF therapy modulates intra-tumoral cytokine levels in MDA-MB-231 human breast tumor xenografts. Mean pg/mg total protein is displayed. N = 3 tumors/group assayed in duplicate at the one and four week time points. Values in *italic* indicate cytokine levels that decreased significantly compared to control; values in bold indicate cytokine levels that increased significantly compared to control, all p<0.01 or p<0.001 by one-way ANOVA, Bonferroni Multiple Comparison Test. n.d., not detected.
(0.05 MB DOC)

Table S3 Anti-VEGF therapy modulates intra-tumoral and serum cytokine levels in 4T1 murine breast tumor xenografts. Mean pg/mg total protein if displayed. N = 3 tumors/group assayed in duplicate at the one and three week time points. Values in *italics* indicate cytokine levels that decreased significantly compared to control; values in bold indicate cytokine levels that increased significantly compared to control, all p<0.01 or p<0.001 by one-way ANOVA, Bonferroni Multiple Comparison Test. n.d., not detected.
(0.05 MB DOC)
