# Supplementary figures and images for: Cytokine Levels Correlate with Immune Cell Infiltration after Anti-VEGF Therapy in Preclinical Mouse Models of Breast Cancer
Source: PLoS One. 2009 Nov 3;4(11):e7669. doi: 10.1371/journal.pone.0007669 (PMC2766251; doi:10.1371/journal.pone.0007669)

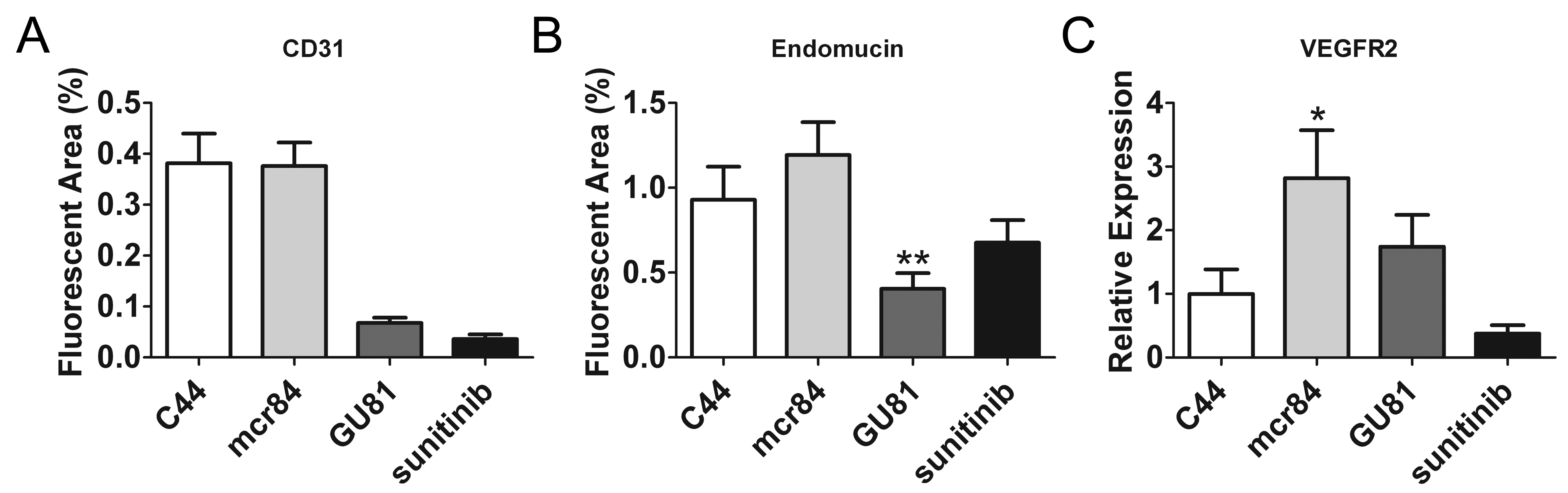

Supplement: Figure S2 — Effect of anti-VEGF therapy on vascular parameters in 4T1 tumors. Mice bearing established orthotopic 4T1 tumors were treated for 3 weeks with control IgG (C44) mcr84 (250 µg ip 2x/week), GU81 (120 µg/day via osmotic pump), or sunitinib (200 µg/day) (n = 4/group). Tumor sections (n = 4) were stained for CD31 (A), endomucin (B) or VEGFR2 (C) by immunofluorescence. Data are displayed as mean±SEM and represents 5 images per tumor and three tumors per group. Total magnification 100X. The mean fluorescent area was determined with Elements software. *, p<0.05; **, p<0.01 vs control by ANOVA. (0.38 MB TIF) [file pone.0007669.s002.tif]

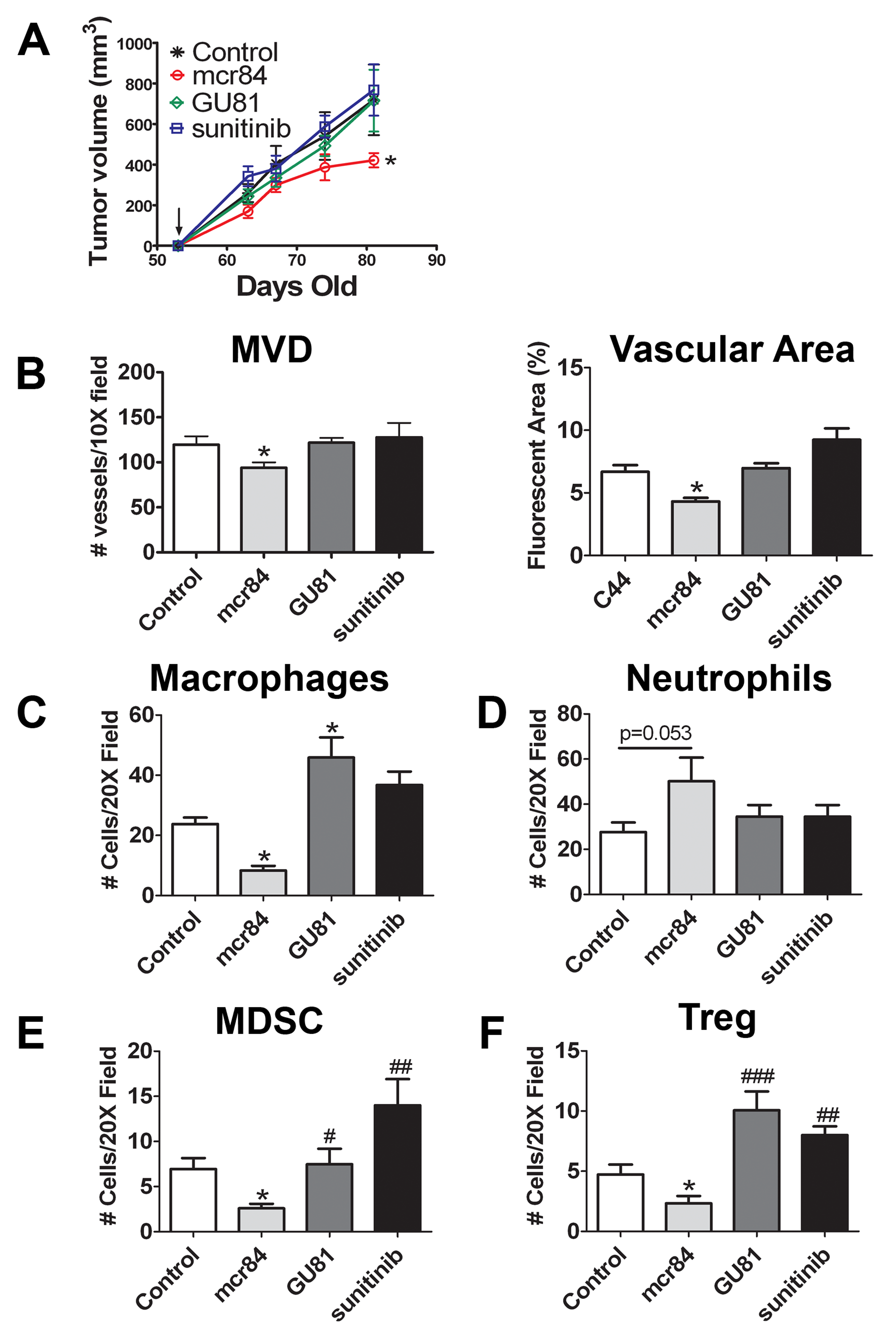

Supplement: Figure S3 — Effect of anti-VEGF therapy on immune cell infiltration in the transgenic MMTV-PyMT breast tumor model. (A) Treatment with 250 µg twice weekly of either control IgG or mcr84, 120 µg daily GU81 or 200 µg daily sunitinib was initiated when transgenic females were 52 days old (arrow) and continued for 4 weeks (n = 5/group). Tumor volumes were measured twice weekly and mean tumor volume +/− SEM is displayed. (B–D) Tumor sections were analyzed by immunofluorescence using MECA-32, an endothelial cell marker (B), macrophages (CD11b+Gr1- cells) (C) and neutrophils (CD11b-Gr1+) (D). (E–F) Tumor sections were evaluated by immunofluorescence for (E) MDSCs, co-localization of CD11b+ and Gr1+ (F) Tregs, co-localization of CD25+and FoxP3+. Data are displayed as mean±SEM and represents 5 images per tumor and three tumors per group. Total magnification, 200X, except for Meca-32 staining (100X). Images were overlayed and using Elements software. *p = 0.05, **p = 0.01, ***p<0.001, ##p = 0.01 vs mcr84. (3.41 MB TIF) [file pone.0007669.s003.tif]
