# Supplementary material for: Cytokine Levels Correlate with Immune Cell Infiltration after Anti-VEGF Therapy in Preclinical Mouse Models of Breast Cancer
Source: PLoS One. 2009 Nov 3;4(11):e7669. doi: 10.1371/journal.pone.0007669 (PMC2766251; doi:10.1371/journal.pone.0007669)
Supplement: Table S1 — 4T1 cells express VEGFR1 but not VEGFR2. RNA isolated from murine (bEnd.3) endothelial cells and 4T1 cells was used for qRT-PCR analysis of VEGFR1 and VEGFR2 and normalized to GAPDH. The mean Ct (cycle threshold) value for each target is displayed. (0.03 MB DOC) [file pone.0007669.s004.doc]

|  | **bEND.3** | **4T1** |
| --- | --- | --- |
| **VEGFR1** | **25.981** | **31.034** |
| **VEGFR2** | **15.79** | **Undet.** |
| **GAPDH** | **14.104** | **15.19** |

**Table S1. 4T1 cells express VEGFR1 but not VEGFR2.** RNA isolated from murine (bEnd.3) endothelial cells and 4T1 cells was used for qRT-PCR analysis of VEGFR1 and VEGFR2 and normalized to GAPDH. The mean *Ct* (cycle threshold) value for each target is displayed.
