# Supplementary material for: Cytokine Levels Correlate with Immune Cell Infiltration after Anti-VEGF Therapy in Preclinical Mouse Models of Breast Cancer
Source: PLoS One. 2009 Nov 3;4(11):e7669. doi: 10.1371/journal.pone.0007669 (PMC2766251; doi:10.1371/journal.pone.0007669)
Supplement: Table S2 — Anti-VEGF therapy modulates intra-tumoral cytokine levels in MDA-MB-231 human breast tumor xenografts. Mean pg/mg total protein is displayed. N = 3 tumors/group assayed in duplicate at the one and four week time points. Values in italic indicate cytokine levels that decreased significantly compared to control; values in bold indicate cytokine levels that increased significantly compared to control, all p<0.01 or p<0.001 by one-way ANOVA, Bonferroni Multiple Comparison Test. n.d., not detected. (0.05 MB DOC) [file pone.0007669.s005.doc]

| **Cytokine** | **Week** | **Control** | **r84** | **bev** | **RAFL-2** | **GU81** | **sunitinib** |
| --- | --- | --- | --- | --- | --- | --- | --- |
| IL1β | 1 | 1.877 | 1.887 | **4.987** | 3.417 | 2.46 | 1.577 |
| IL1β | 4 | 2.195 | 2.044 | 3.56 | 0.8987 | **14.12** | 1.876 |
| IL10 | 1 | 0.213 | 0.282 | 0.888 | **1.944** | 1.672 | 1.422 |
| IL10 | 4 | 3.124 | *1.011* | 3.967 | 1.415 | 1.438 | *1.064* |
| IL12 | 1 | 167.8 | 153 | 220.2 | **301.7** | 219.1 | 145.4 |
| IL12 | 4 | 277.6 | 233.3 | 245.1 | 88.2 | 270.2 | 189.9 |
| IL2 | 1 | n.d. | n.d. | n.d. | n.d. | n.d. | n.d. |
| IL2 | 4 | 0.277 | n.d. | 0.329 | n.d. | n.d. | n.d. |
| IL4 | 1 | n.d. | n.d. | n.d. | n.d. | n.d. | n.d. |
| IL4 | 4 | n.d. | n.d. | n.d. | n.d. | n.d. | n.d. |
| IL5 | 1 | n.d. | n.d. | 0.125 | **0.379** | 0.265 | 0.193 |
| IL5 | 4 | 0.478 | 0.18 | 0.477 | 0.152 | 0.259 | 0.213 |
| CXCL1 | 1 | 10.51 | 9.62 | 31.6 | 10.25 | 12.81 | 10.76 |
| CXCL1 | 4 | 30.96 | 33.78 | 32.77 | *12.69* | 44.05 | 56.86 |
| IFNγ | 1 | n.d. | n.d. | n.d. | n.d. | n.d. | n.d. |
| IFNγ | 4 | 0.435 | 0.181 | 0.291 | n.d**.** | 0.082 | *0.055* |
| TNFα | 1 | n.d. | n.d. | n.d. | n.d. | n.d. | n.d. |
| TNFα | 4 | n.d. | n.d. | n.d. | n.d. | n.d. | n.d. |
| Total MMP-9 |  | 142.6 | 175.5 | 204.2 | *90.9* | 162.5 | 99.02 |
| active MMP-9 |  | 89.15 | 109.4 | 111 | *48.07* | 85.61 | 63.79 |

**Table S2. Anti-VEGF therapy modulates intra-tumoral cytokine levels in MDA-MB-231 human breast tumor xenografts.** Mean pg/mg total protein is displayed. N=3 tumors/group assayed in duplicate at the one and four week time points. Values in *italic* indicate cytokine levels that decreased significantly compared to control; values in **bold** indicate cytokine levels that increased significantly compared to control, all p<0.01 or p<0.001 by one-way ANOVA, Bonferroni Multiple Comparison Test. n.d., not detected.
