# Supplementary material for: Cytokine Levels Correlate with Immune Cell Infiltration after Anti-VEGF Therapy in Preclinical Mouse Models of Breast Cancer
Source: PLoS One. 2009 Nov 3;4(11):e7669. doi: 10.1371/journal.pone.0007669 (PMC2766251; doi:10.1371/journal.pone.0007669)
Supplement: Table S3 — Anti-VEGF therapy modulates intra-tumoral and serum cytokine levels in 4T1 murine breast tumor xenografts. Mean pg/mg total protein if displayed. N = 3 tumors/group assayed in duplicate at the one and three week time points. Values in italics indicate cytokine levels that decreased significantly compared to control; values in bold indicate cytokine levels that increased significantly compared to control, all p<0.01 or p<0.001 by one-way ANOVA, Bonferroni Multiple Comparison Test. n.d., not detected. (0.05 MB DOC) [file pone.0007669.s006.doc]

| **Cytokine** | **Week** | **Control** | **mcr84** | **GU81** | **sunitinib** |
| --- | --- | --- | --- | --- | --- |
| IL1β | 1 | 52.16 | 37.82 | 21.16 | 27.88 |
| IL1β | 3 | 41.69 | **102.9** | 64.31 | 79.41 |
| IL10 | 1 | 11.67 | 8.544 | *7.481* | 7.096 |
| IL10 | 3 | 6.268 | 7.547 | 3.961 | 8.011 |
| IL12 | 1 | 422.4 | 527.1 | 198.8 | 286.6 |
| IL12 | 3 | 191.8 | 545 | **816.7** | 172.4 |
| IL2 | 1 | 2.209 | 1.77 | 1.473 | 1.572 |
| IL2 | 3 | 1.581 | 1.555 | 1.771 | 1.748 |
| IL4 | 1 | 1.696 | 1.38 | *1.161* | 1.085 |
| IL4 | 3 | 1.147 | 1.159 | 0.8129 | 1.365 |
| IL5 | 1 | 1.189 | 1.031 | 0.9213 | 0.8637 |
| IL5 | 3 | 1.239 | 1.183 | 1.175 | 1.222 |
| IL6 | 1 | 31.19 | *12.74* | 10.4 | 13.69 |
| IL6 | 3 | 8.92 | **20.69** | 8.84 | 10.1 |
| CXCL1 | 1 | 209.7 | 119 | *78.14* | 134.2 |
| CXCL1 | 3 | 137.4 | **395.5** | 286.4 | 323.2 |
| IFNγ | 1 | 2.363 | 1.57 | *1.02* | 1.426 |
| IFNγ | 3 | 2.205 | 1.625 | 0.9552 | 1.24 |
| TNFα | 1 | 6.77 | 6.003 | 4.917 | 5.634 |
| TNFα | 3 | 5.519 | 4.54 | 5.376 | 5.486 |
| Active TGFβ | 1 | 250.6 | 268.8 | 312.6 | 233.6 |
| Active TGFβ | 3 | 252.5 | 312.3 | 175.9 | 133.4 |
| serum IL6 | 3 | 8.844 | **35.22** | 32.04 | 52.60 |

**Table S3. Anti-VEGF therapy modulates intra-tumoral and serum cytokine levels in 4T1 murine breast tumor xenografts^a^.**Mean pg/mg total protein if displayed. N=3 tumors/group assayed in duplicate at the one and three week time points. Values in *italics* indicate cytokine levels that decreased significantly compared to control; values in **bold** indicate cytokine levels that increased significantly compared to control, all p<0.01 or p<0.001 by one-way ANOVA, Bonferroni Multiple Comparison Test. n.d., not detected.
